# Phytochemical Analysis and Modulation of Antibiotic Activity by *Luehea paniculata* Mart. & Zucc. (Malvaceae) in Multiresistant Clinical Isolates of *Candida* Spp.

**DOI:** 10.1155/2015/807670

**Published:** 2015-02-22

**Authors:** João T. Calixto Júnior, Selene M. Morais, Clécio G. Martins, Larissa G. Vieira, Maria Flaviana B. Morais-Braga, Joara N. P. Carneiro, Antonio J. P. Machado, Irwin R. A. Menezes, Saulo R. Tintino, Henrique D. M. Coutinho

**Affiliations:** ^1^Laboratório de Produtos Naturais, Universidade Estadual do Ceará (UECE), 60740-000 Fortaleza, CE, Brazil; ^2^Rede Nordeste de Biotecnologia (RENORBIO), 60740-000 Fortaleza, CE, Brazil; ^3^Faculdade de Juazeiro do Nort (FJN), 63010-210 Juazeiro do Norte, CE, Brazil; ^4^Laboratório de Microbiologia e Biologia Molecular, Universidade Regional do Cariri (URCA), 63105-000 Crato, CE, Brazil; ^5^Laboratório de Farmacologia e Química Molecular, Universidade Regional do Cariri (URCA), 63105-000 Crato, CE, Brazil

## Abstract

The high incidence of fungal infections has led to the continuous search for new drugs. Extracts of *Luehea paniculata*, a tree of multiple medicinal uses, were evaluated for anti-*Candida* activity, as well as its modulator potential of the Fluconazole antibiotic. Chemical prospecting of ethanol extracts of leaf and bark was carried out, the quantification of total phenols and flavonoids, characterized by the HPLC-DAD technique. The rosmarinic acid and the vitexin flavonoid were observed as major constituents in ELELP and ESWELP, respectively. Antioxidant activity was also evaluated by the method of scavenging the free radical DPPH, and quercetin was used as standard, obtaining IC_50_ values: 0.341 (mg/mL) for ELELP and 0.235 (mg/mL) for ESWELP. The microdilution assay was performed for antifungal activity against strains of *Candida albicans, C. krusei*, and *C. tropicalis* and showed minimum inhibitory concentrations values ≥1024 *μ*g/mL. In the modulator action of extracts on Fluconazole against multiresistant clinical isolates of *Candida* (subinhibitory concentration minimum of 128 *μ*g/mL), a significant synergism was observed, indicating that the extracts potentiated the antifungal effect against *C. tropicalis*, where antioxidant flavonoids could be responsible. This is the first report about modifying activity of the antibiotic action of a species of the genus *Luehea*.

## 1. Introduction 

Northeastern Brazil presents a huge variety of plants, many of which display biological activities [1]. Essential oils from Caatinga biome plants as* Lippia sidoides* and* Croton *spp. species have shown antifungal activity against* Candida* spp. and* Microsporum canis* [2, 3].

Opportunistic fungal infections have increased in recent years, mostly occurring in hospital settings and in immunocompromised individuals. Yeasts of the genus* Candida* caused the most common, superficial, and invasive infections [4]. These infections comprise a big problem nowadays, since they are increasingly common in immunosuppressive diseases, in which these fungi turn into opportunistic pathogens potentially responsible for high mortality [5, 6]. The mortality rate even after drug treatment is high, ranging from 40 to 60% [7]. The incidence of fungal infections has led to a continuous search for new drugs, which besides having a biocide effect would entail no side effects. This is configured as a major challenge, since fungi are, like humans, eukaryotes and as a result of the similarity between the cells, many commercial drugs have caused cytotoxicity in the liver and kidneys of the host [8].

According to Sorg [9], diseases such as cancer, emphysema, cirrhosis, arthritis, and inflammation, in addition to the aging process itself, are related to free radicals. Elejalde Guerra [10] explained that antioxidant therapy and diet enriched with antioxidants appear to prevent or at least mitigate the organic deterioration by oxidative stress. It can be observed that in recent decades there has been growth of scientific research in this area, involving the effect of crude extracts, purified fractions, or isolated components, highlighting phenolic compounds that, in many studies, have shown this activity [1, 11]. Therefore, antioxidant activity, which protects the live organism against deterioration, could play an important role for an antifungal agent.

Malvaceae are a family consisting of herbs, subshrubs, shrubs, lianas, and small and large trees, with about 250 genera and 4200 species, and in Brazil, there are about 80 genera and 400 species [12]. According to the List of Species of Flora of Brazil, 69 genera and 754 species are pointed out [13], distributed in 30 genera and 393 taxa of the Malvaceae subfamily [14]. The genus* Luehea* is one of 252 genera of the Malvaceae family. The leaves of* Luehea* are marketed as herbal against dysentery, leucorrhoea, rheumatism, gonorrhea, and tumors; the infusion of the flowers is used against bronchitis and the root is depurative [15]. Braga [16] pointed out the astringency of the barks of* L. speciosa* Willd. (botanical synonym of* L. divaricata*) and also recorded the occurrence of the species* L. candicans* Mart. et Zucc. (*Luehea uniflora* St. Hil.) and* L. paniculata* Mart. presenting the same use as the first named.

In this context, the aim of this work was to investigate the antifungal effect of ethanol extract from different parts of* Luehea paniculata *Mart. & Zucc., a medicinal tree of multiple effects, individually and in combination with a commercial drug, against clinical isolates of multidrug-resistant strains of* Candida*. An evaluation of free radical-scavenging activity and phytochemical analysis of the extracts were also performed in order to relate the existence of phenolic compounds with activities determined in this study.

## 2. Material and Methods 

### 2.1. Place of Collection of the Plant Material and Obtaining Extracts

Leaves and sapwood of three specimens of* L. paniculata* were collected on November 19, 2012 (dry season), around 17:30 hours, from the Cerrado located in the center of Caatinga area, Lavras da Mangabeira city, central Mesoregion in South of Ceará, Northeastern Brazil.

The area is characterized as a vegetation relic [17], presenting itself in deep soil on a tabular relief on long dissected surfaces with penetration of the savannah flora. The patch of savannah in question is located in hills of 280 to 600 m in height, at the following coordinates: 06°72′2432′′S and 38°97′7396′′W. The botanical material was collected and deposited in the Prisco Bezerra Herbarium of the Federal University of Ceará, under number 54641.

The plant material was dried at 60°C, weighed (Table 1), and subjected to maceration with ethanol PA for seven days. After this period, filtration and concentration of the extract were made distilling all the solvent on a rotary evaporator under reduced pressure (40 rpm at 60°C) and then in a water bath at 60°C, obtaining the crude extracts.

### 2.2. Evaluation of Antioxidant Activity

Using the method developed by Yepez et al. [18], 0.1 mL of methanol solution of sample (10 000–1 ppm) was added to a test tube containing 3.9 mL of 6.5 10^−5^ M of 1,1-diphenyl-2-picryl-hydrazyl (DPPH) methanol solution. The test was performed in triplicate and the results were considered positive if the absorbance decreased with time. After 1.0 h, the UV absorbance of the mixture was measured at 515 nm. To calculate the sample potential for DPPH inhibition in terms of percentage (IP%), the following equation was used:

(1) $$\begin{array}{c} \text{IP}=\frac{A_{\text{DPPH}}-A_{\text{Sample}}}{A_{\text{DPPH}}}\times 100. \end{array}$$

The inhibitory potential (%) was applied in the Origin 7.0 statistic program to calculate the 50% inhibitory concentration (IC_50_), where *A*_DPPH_ is the absorbance of the DPPH solution and *A*_Sample_ is the absorbance of the solution containing the extract at a particular concentration.

### 2.3. Quantification of Flavonoids and Total Phenols in the Extracts

The extracts were subjected to determination of flavonoids according to the methodology proposed by Funari and Ferro [19]. A stock solution was prepared by adding to a round bottom flask 1.2 mg of extract and completing the volume to 10 mL with methanol (70%). 2 mL of this solution was transferred to another 10 mL flask. 1 mL of aluminum chloride (AlCl_3_) chloride 15% was added and the volume was completed to 10 mL with methanol (70%). The solution was allowed to stand for 30 minutes and then the spectrophotometric reading was performed at 425 nm. This test was performed in triplicate.

Total phenolics content was determined by the spectrophotometric method using the Folin-Ciocalteu reagent and gallic acid as reference standard [20]. Ethanol extracts (7.5 mg) were dissolved in methanol and transferred to a 25 mL volumetric flask and final volume was completed with methanol. An aliquot of 100 *μ*L of the latter solution was shaken with 500 *μ*L of Folin-Ciocalteu reagent and 6 mL of distilled water for 1 min; after this time 2 mL of 15% Na_2_CO_3_ was added to the mixture and stirred for 30 s. Finally, the solution was settled to 10 mL volume with distilled water. After 2 hours of incubation, the absorbance of the samples was measured at 750 nm.

### 2.4. Chemical, Apparatus, and General Procedures

All chemicals were of analytical grade. Methanol, formic acid, gallic acid, chlorogenic acid, and rosmarinic acid were purchased from Merck (Darmstadt, Germany). Vitexin, luteolin, and rutin were acquired from Sigma Chemical Co. (St. Louis, MO, USA). High performance liquid chromatography (HPLC-DAD) was performed with a Shimadzu Prominence Auto Sampler (SIL-20A) HPLC system (Shimadzu, Kyoto, Japan), equipped with Shimadzu LC-20AT reciprocating pumps connected to a DGU 20A5 degasser with a CBM 20A integrator, SPD-M20A diode array detector, and LC solution 1.22 SP1 software.

### 2.5. Quantification of Compounds by HPLC-DAD

Reverse phase chromatographic analyses were carried out under gradient conditions using C_18_ column (4.6 mm × 150 mm) packed with 5 *μ*m diameter particles; the mobile phase was water containing 1% formic acid (A) and methanol (B), and the composition gradient was 12% of B until 10 min and was changed to obtain 20%, 30%, 50%, 60%, 70%, 20%, and 10% of B at 20, 30, 40, 50, 60, 70, and 80 minutes, respectively, following the method described by Boligon et al. [21] with slight modifications.* L. paniculata* ethanolic extracts and mobile phase were filtered through 0.45 *μ*m membrane filter (Millipore) and then degassed by ultrasonic bath prior to use and the extracts were analyzed at a concentration of 10 mg/mL. The flow rate was 0.8 mL/min and injection volume was 40 *μ*L and the wavelength was 271 nm for gallic acid, 327 nm for chlorogenic acid and rosmarinic acid, 337 nm for vitexin, and 365 nm for luteolin and rutin. All the samples and mobile phase were filtered through 0.45 *μ*m membrane filter (Millipore) and then degassed by ultrasonic bath prior to use. Stock solutions of standards references were prepared in the HPLC mobile phase at a concentration range of 0.025–0.250 mg/mL for luteolin, vitexin, and rutin and 0.030–0.350 mg/mL for gallic acid, chlorogenic acid, and rosmarinic acid. Chromatography peaks were confirmed by comparing their retention time with those of reference standards and by DAD spectra (200 to 500 nm). Calibration curve for gallic acid is  *Y* = 12509*x* + 1354.8 (*r* = 0.9996); chlorogenic acid, *Y* = 11948*x* + 1256.5 (*r* = 0.9999); vitexin, *Y* = 12647*x* + 1346.9 (*r* = 0.9997); rosmarinic acid, *Y* = 13476*x* + 1197.6 (*r* = 0.9999); rutin, *Y* = 12706*x* + 1384.5 (*r* = 0.9999); and luteolin, *Y* = 11865*x* + 1259.6 (*r* = 0.9998). All chromatography operations were carried out at ambient temperature and in triplicate.

The limit of detection (LOD) and limit of quantification (LOQ) were calculated based on the standard deviation of the responses and the slope using three independent analytical curves. LOD and LOQ were calculated as 3.3 and 10*σ*/*S*, respectively, where *σ* is the standard deviation of the response and *S* is the slope of the calibration curve [22].

### 2.6. Antifungal Assay: Preparation of the Initial Solution

The preparation of the starting solution of the samples was made by weighing 10 mg of extracts and diluting them in 1 mL of dimethyl sulfoxide (DMSO, Merck, Darmstadt, Germany) to achieve an initial concentration. From this concentration, dilution was made in sterile distilled water to achieve a concentration of 2048 mg/mL (solution test).

### 2.7. Strains, Drugs, and Culture Media Used

The microorganisms used in sensitivity to natural products testing were obtained from the Mycology Laboratory (LM) of the Federal University of Paraíba (UFPB) and the Laboratory of Microbiology and Molecular Biology (LMBM), the Regional University of Cariri (URCA). Three standard yeast* Candida albicans* (CA 77),* Candida krusei* (CK 01), and* Candida tropicalis* (CT 23) strains were used; besides these, collections of multiresistant clinical isolates of the three species of fungi cited were used: CA 40006, CK 40095, and CT 40042. The Fluconazole antibiotic was used as the reference drug in the modulation of the commercial drug test.

The yeasts were first grown on solid medium Sabouraud dextrose agar (SDA) and then transferred to test tubes containing 5 mL of sterile saline. The concentration of the strains was diluted in 0.9% saline solution until a concentration of 10^5^ UFC/mL was obtained and compared with the nephelometric McFarland scale [23]. This procedure provided a standard yeast suspension containing 1 × 10^5^ cells per mL [24]. In microdilution tests, Sabouraud dextrose broth (CSD) was the medium used.

### 2.8. Test Minimum Inhibitory Concentration (MIC)

Eppendorf tubes were prepared for distribution in microdilution plate each containing 1.5 mL of solution containing 1350 to 150 *μ*L CSD *μ*L of fungal suspension. The plate was completed by adding 100 *μ*L of this solution to each well (96-well plates) and then proceeding to serial microdilution with a solution of 100 *μ*L of the natural product (extract), varying concentrations 1024–8 mg/mL, with the last well for growth control. The plates were put inside the incubator for 24 hours at 35°C [25], and thereafter readings were taken in a spectrophotometric ELISA (Thermoplate) with a wavelength of 630 nm device. The MIC was defined as the lowest concentration at which no growth was observed. All tests were performed in triplicate.

### 2.9. Test Modulating Action of Antifungal

To investigate the effect of the modulator antifungal action of the front lines, the method proposed by Coutinho et al. [26] was used, where the solution of the extract was tested at subinhibitory concentration (MIC/8). Eppendorf tubes were prepared each containing 1.5 mL of the culture medium containing CSD, 150 *μ*L of the fungal suspension and the natural product concentration MIC/8. For the control, Eppendorf tubes with 1.5 mL of solution containing 1350 *μ*L and 150 *μ*L CSD suspension of microorganisms were prepared. The ELISA plate was completed by adding 100 *μ*L of this solution to each well. Then, 100 *μ*L of drug (antifungal agent) was mixed well to the first, proceeding in the broth microdilution series until the penultimate cavity. The concentrations of antifungal gradually decreased from 1024 to 1 mL.

### 2.10. Statistical Analysis

The determinations were made in triplicate and the results were normalized by calculating the geometric mean. The results were compared using analysis of variance (ANOVA) and the comparison between the geometric means was performed according to Tukey's test, which was considered significant when *P* < 0.05 [27].

## 3. Results 

### 3.1. Antioxidant Activity and Relation with Phenolic Compounds

**Figure 1:**
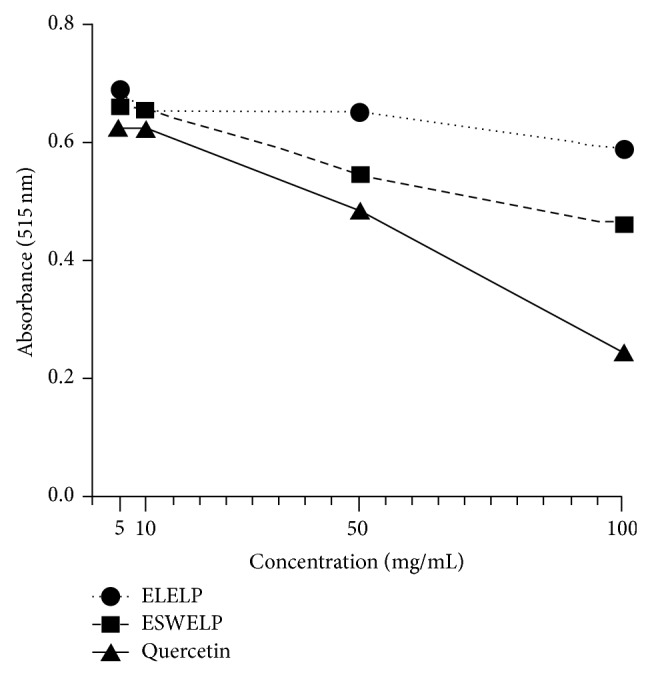
Antioxidant activity in different concentrations of* Luehea paniculata* extracts. ELELP:* L. paniculata* ethanolic leaf extract; ESWELP:* L. paniculata* ethanolic extract of sapwood.

The results of the antioxidant activity, content of total phenols and flavonoids, and IC_50_ values (concentration of the sample can sequester 50% of the DPPH radicals) determined for extracts of* L. paniculata* and the positive control (quercetin) are shown in Table 2 and Figure 1. The content of total phenols and of flavonoids was higher in the leaf extract than in the extract of sapwood.

### 3.2. HPLC Analysis

**Figure 2:**
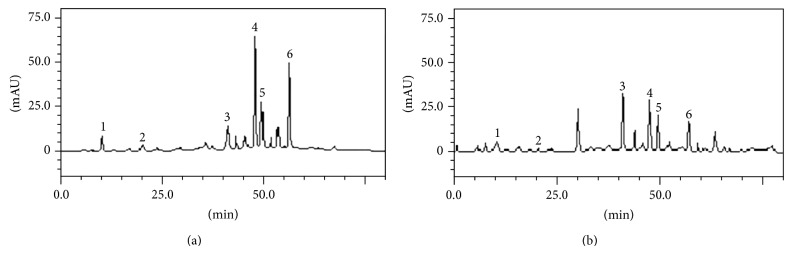
Representative high performance liquid chromatography profile of* L. paniculata* ethanolic extracts ((a) EEFLP; (b) EEECLP). Gallic acid (peak 1), chlorogenic acid (peak 2), vitexin (peak 3), rosmarinic acid (peak 4), rutin (peak 5), and luteolin (peak 6).

HPLC fingerprinting of* L. paniculata* ethanolic extracts revealed the presence of the gallic acid (*t*_*R*_ = 0.37 min; peak 1), chlorogenic acid (*t*_*R*_ = 20.19 min; peak 2), vitexin (*t*_*R*_ = 41.78 min; peak 3), rosmarinic acid (*t*_*R*_ = 48.15; peak 4), rutin (*t*_*R*_ = 49.87 min; peak 5), and luteolin (*t*_*R*_ = 57.12 min; peak 6) (Figure 2 and Table 3).

### 3.3. Anti-*Candida* Activity and Modulation of the Antibiotic

**Figure 3:**
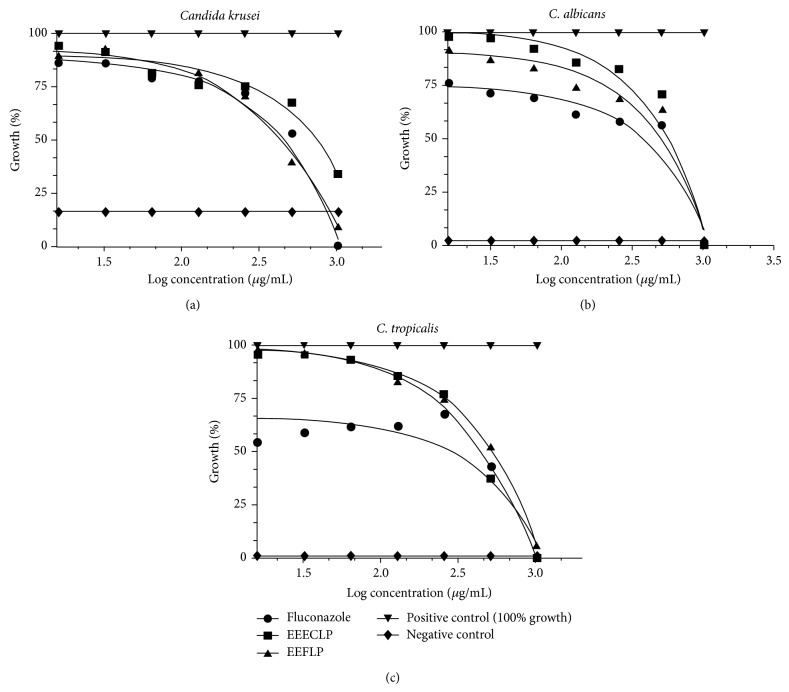
Values of MIC of natural products of* L. paniculata* and antibiotic Fluconazole next to the strains of* Candida* graphics. ELELP:* L. paniculata* ethanolic leaf extract; ESWELP:* L. paniculata *ethanolic extract of sapwood.

In this study, the* L. paniculata* ethanolic leaf extract (ELELP) tested against six strains of* Candida *demonstrated clinically irrelevant activity for the yeast* C. krusei,* CK 40095 and CK 01 (MIC ≥ 1024). In the other strains (CA 77, CA 40006, CT 23, and CT 40042) there was an insignificant effect, and MIC 1024 *μ*g/mL was also observed. The ESWELP presented MIC 1024 *μ*g/mL for all strains tested, being considered of inhibitory effect without relevance to the yeasts (Figure 3).

### 3.4. Statistical Analysis

Statistically significant differences by Tukey's test (*P* > 0.05) for both extracts were observed for the amounts related to growth inhibition in* C. albicans* and* C. tropicalis* compared with the curve of death expressed by Fluconazole, indicating irrelevant effects when used alone against the yeast strains. Relative to* C. krusei* significant differences (*P* > 0.05) were not observed, having a similar rate of growth inhibition to antifungal, although neither Fluconazole nor natural products exhibit clinically relevant activity, whereas MIC value is ≥1024 *μ*g/mL for both. The proximity expressed in the graph between the growth rates in lower concentrations cannot be substantiated by relevant clinical activity of both natural products of* L. paniculata*, as Fluconazole for* C. krusei*.

**Figure 4:**
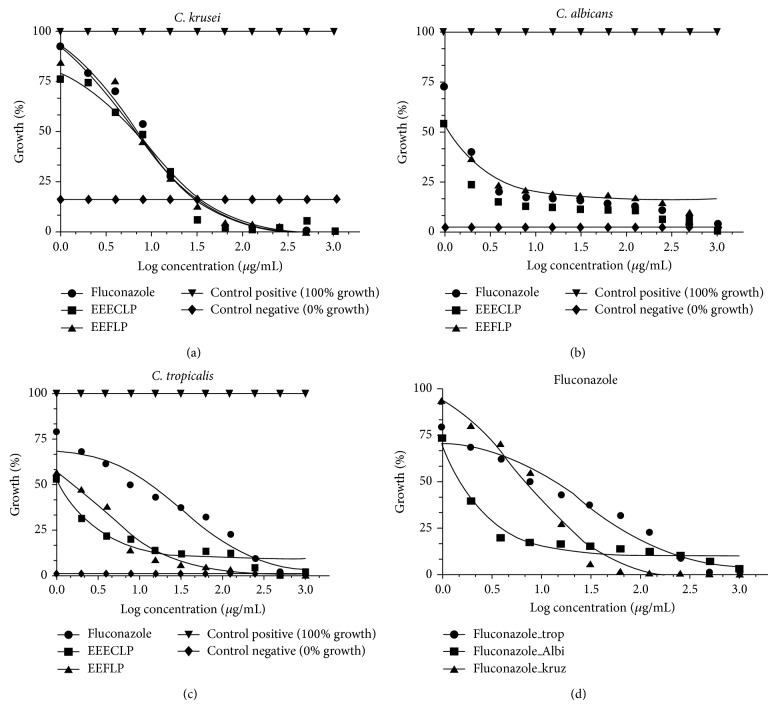
Modulatory effect of natural products of* L. paniculata* in association with antifungal Fluconazole next to multiresistant strains of* Candida* graphics. ELELP:* L. paniculata* ethanolic leaf extract; ESWELP:* L. paniculata* ethanolic extract of sapwood.

This work is the first recorded piece of research on the potentialization of Fluconazole using natural products from* L. paniculata*. In the tests carried out to measure this modulating effect, it was observed that natural products do indeed potentialize the effect of the antifungal agent against three strains of* Candida*, in particular* C. tropicalis* and* C. albicans*, and that there was, therefore, synergism when the extracts were combined with Fluconazole (Figure 4).

Using the Tukey test (*P* > 0.05), statistically significant differences were observed between the absorbance values of *x* growth of* C. tropicalis*, indicating that the action of Fluconazole alone was much lower than that in association with* L. paniculata* extracts. Significant *P* < 0.0001 (ANOVA) statistical values were also observed for the modulatory action of* C. tropicalis*. For* C. albicans,* there was a significant difference (*P* > 0.05) between the results of Fluconazole and* L. paniculata *ethanolic extract of sapwood (ESWELP). However, the same difference was not observed between the results of Fluconazole and ELELP, indicating that this extract did not alter the effect of the antibiotic.* C. krusei* and no statistical difference was observed (*P* > 0.05).

## 4. Discussions 

Phenolic compounds and some of their derivatives are considered effective agents in preventing oxidation and inhibiting or eliminating free radicals that affect organ systems. Many of them are very good at radical scavenging and most of these are found as esters or glycosides, which are soluble in water and polar organic solvents [28]. Phytochemical studies with other species of the genus* Luehea* collected in other regions of Brazil have shown the presence of phenolic compounds, especially flavonoids [15, 29–32].

The fact that the content of total phenols and flavonoids is higher in the leaf extract than in the extract of the sapwood is related to various environmental factors that influence the production of flavonoids in plants, such as temperature, nutrition, injury, radiation quality, and the metabolism of sugar and nitrogen [33, 34], since leaves are more exposed to environmental stress. For Oliveira [35], solar radiation is a factor that is usually related to quantitative variation. Some studies have shown a quantitative increase of flavonoids in organs exposed to light, compared to those subjected to lower.

The ethanol extract of sapwood (ESWELP) displayed an antioxidant activity value (IC_50_ = 0.235 mg/mL), similar to the standard quercetin (IC_50_ = 0.203 mg/mL), mainly due to the presence of the flavonoids vitexin, rosmarinic acid, rutin, and luteolin as characterized by HPLC comparison with correspondent standards. The ELELP also resulted in optimal antioxidant activity, close to the standard (IC_50_ = 0.341 mg/mL), relating this activity to flavonoids especially rosmarinic acid, luteolin, and rutin. Good antioxidant activity of the ethanol extract of leaves of other species of the genus,* L. divaricata,* has been cited by Müller [36]. Using the DPPH method, the author observed the crude extract and the fractions ethyl acetate and butanol with antioxidant activity similar to quercetin.

According to Afanas'ev et al. [37] who investigated the antioxidant activity of rutin, this flavonoid has a therapeutic action in diseases involving free radicals and is not considered toxic. Several other activities of rutin have been elucidated such as antihyperlipidemic activity [38], suppression of cellular immunity [39], and anti-inflammatory [40] and anticonvulsant effect in rats [41]. Their effectiveness in the treatment of diseases caused by* Candida albicans* was investigated and confirmed by Han [42]. Phenolic compounds including flavonoids have demonstrated their therapeutic potential as anti-inflammatory, antifungal, antimicrobial, antioxidant, and wound healing agents [43]. Rosmarinic acid also has significant antioxidant activity [44], reducing the body numerous deleterious events such as the formation of reactive oxygen species, lipid peroxidation, and DNA fragmentation [45, 46].

Akio Tanaka et al. [15] observed that* Candida albicans, C. krusei, C. parapsilosis,* and* C*.* tropicalis* were resistant to extracts of other species of the genus,* Luehea divaricata*. Coelho de Souza et al. [47], in an ethnopharmacological study, evaluated the antimicrobial potential of some plants in common use in the Rio Grande do Sul State, Brazil, among them* L. divaricata*. Using the agar diffusion method, the authors found that the methanol extract of leaves showed no activity against* C. albicans*. The antifungal activity of* L. divaricata* was evaluated by Zacchino et al. [48]. As a result, the authors reported a moderate action of dichloromethane extract in inhibiting the growth of fungal hyphae of dermatophytes.

Silva [49] studying* L. candicans* in the region of Puerto Rico, Paraná State, Brazil, evaluated antifungal activity of crude extracts, fractions, and isolated compounds. The crude extract of the leaves showed antifungal activity against* C. krusei* strain at a concentration of 125 mg/mL (CMF) and the fungistatic concentration of 62.5 mg/mL (MIC). Marques et al. [50] studied 23 species of Cerrado another vegetation type region in Brazil (Midwest) and found, in relation to* L. paniculata*, a good inhibitory of microorganisms, among them, strains of* Candida*.

Literature data show that, of the constituents found in higher percentages, only quercetin does not present antifungal activity when tested against standard strains of* C. albicans* ATCC 40227 and* C. krusei* ATCC 6538 [6, 51]. Rutin, when tested against standard strains of* C. albicans, C. krusei,* and* C*.* tropicalis* (ATCC 13803), demonstrated similar action to the diluent DMSO [6, 52].

The potentiation of Fluconazole activity reported in this study, mainly observed for* C. tropicalis* (both extracts) and* C. albicans* (ethanol extract of sapwood), can be attributed to the synergistic activity of flavonoid constituents present in extracts. This can be inferred in view of the proven antimicrobial activity [53] and antibiotics modulator [54–56] by flavonoids. There are other studies, which demonstrate Fluconazole modulator activity of natural products. Endo et al. [57] reported potent antifungal activity of extracts and pure compound isolated from* Punica granatum *and synergism with fluconazole against* Candida albicans*. The combination of these may alter the membrane permeability of the fungal strains, favoring the intracellular passage of the antifungal, which inhibits the synthesis of ergosterol, causing an increase in death of the microorganisms at a lower concentration. According to Wynn et al. [58], the mechanism of action of azoles such as Fluconazole is the inhibition of ergosterol biosynthesis by interaction with the enzyme lanosterol demethylase, responsible for the conversion of lanosterol to ergosterol, an essential component of the fungal membrane. Inhibition of ergosterol synthesis can cause changes in permeability of the plasma membrane and inhibition of proliferation at all stages of the life cycle of the fungus [59].

The combination of Fluconazole with natural products can be an alternative way of minimizing side effects of the antibiotic, since it leads to a significant synergistic effect, reducing the MIC of the antifungal agent, thus reducing the dose which is necessary for its therapeutic use [56].

The mechanisms by which the extracts can interfere with the growth of microorganisms are varied and may in part be related to the chemical nature of some of their components [27]. As a result, they may show greater interaction with the lipid bilayer of the cellular membrane, which affects the respiratory chain and energy production [60], or even provide increased permeability of the cell to antibiotics, leading to the disruption of cellular activity [61, 62]. The use of extracts as antimicrobial agents has a low risk of increased microbial resistance to their action, because they are complex mixtures, providing greater difficulties for microbial adaptability [63].

Despite the search for new substances in plant extracts through isolation and identification, some results seem to be related to the combination of the compounds contained in these complex mixtures, which characterize these extracts [27]. Veras et al. [51] suggest that quercetin and isoquercitrin, when tested alone, had no significant effect, demonstrating an antagonistic effect on the results of modulation antibiotic. Coutinho et al. [26, 54] stated that these mechanisms of action may be related to the combination of antibiotics with extracts of a subinhibitory concentration added directly to the culture medium.

This strategy is called “herbal shotgun” or “synergistic multieffect targeting” and refers to plants and the use of drugs in a combined approach using single or multiple extracts, which can affect not only a single target but also several different therapeutic compounds which act together in a synergistic or antagonistic manner. Not only does this procedure result from the combination of extracts, but also it is due to combinations of natural and synthetic products or antibiotics [55, 64, 65].

A final approach in relation to antioxidant action of phenolic compounds is due to the mechanism of modulating action of extracts on bacterial antibiotic susceptibility which probably involves antioxidant, preferentially iron-chelating, or prooxidant properties of polyphenols [66].

## 5. Conclusions 

Chemical analysis performed on the natural products of* L. paniculata* revealed the presence of flavonoids such as rutin and vitexin and phenolic acids such as rosmarinic acid, justifying the significant values in the evaluation of antioxidant potential. The results of this study also imply that stem bark and leaf extracts of* L. paniculata* have no clinically relevant antifungal activity; however, in combination with an antibiotic for evaluating the interference of the fungal resistance profile, a significant synergistic effect for* C. tropicalis* is indicated, representing interesting alternative efforts in combating candidiasis.

## Figures and Tables

**Table 1:** Dry weight and yield of the crude ethanol extracts of *L*. *paniculata*.

Natural product	Solvent	Dry matter	Crude extract	Extract yields
ELELP	Ethanol	595 g	61.649 g	10.36%
ESWELP	Ethanol	195 g	36.621 g	18.78%

ELELP: ethanol extract of leaf of* L*. *paniculata*; ESWELP: ethanolextract of sapwood of *L*. *paniculata*.

**Table 2:** Antiradical activity (IC_50_) and total phenols and flavonoids in the extracts of *L*. *paniculata*.

Extracts	Phenolic compounds content (mg EAG g^−1^ of extract)	Flavonoids (mg EAG g^−1^ of extract)	IC_50_ (mg/mL^−1^)	*R* ^2^
ELELP	171.036 ± 0.0143	63.123 ± 0.0311	0.341	0.989
ESWELP	89.021 ± 0.0098	3.001 ± 0.0059	0.235	0.986
Quercetin	—	—	0.203	0.952

—: substance not subjected to the test; EAG: gallic acid equivalent; IC_50_: concentration of sample able to sequester 50% of DPPH radicals; *R* ^2^: coefficient of determination; ELELP: ethanolic leaf extract of *L*. *paniculata*; ESWELP: ethanolic extract of sapwood of *L*. *paniculata*.

**Table 3:** Phenolics and flavonoids composition of *L*. *paniculata* ethanolic extracts.

Compounds	Leaves		Sapwood		LOD	LOQ
	mg/g	%	mg/g	%	*μ*g/mL	*μ*g/mL
Gallic acid	1.14 ± 0.03^a^	0.11	1.11 ± 0.01^a^	0.11	0.017	0.056
Chlorogenic acid	0.39 ± 0.01^b^	0.03	0.09 ± 0.02^b^	0	0.026	0.090
Vitexin	2.37 ± 0.02^c^	0.23	4.98 ± 0.01^c^	0.49	0.032	0.105
Rosmarinic acid	10.06 ± 0.01^d^	1	4.16 ± 0.01^d^	0.41	0.028	0.096
Rutin	4.27 ± 0.01^e^	0.42	2.93 ± 0.02^e^	0.29	0.009	0.034
Luteolin	7.95 ± 0.03^f^	0.79	2.85 ± 0.01^e^	0.28	0.015	0.049

Results are expressed as mean ± standard deviations (SD) of three determinations. Averages followed by different letters differ by Tukey's test at *P* < 0.05. Limit of detection (LOD) and limit of quantification (LOQ).
